# Pancreatitis, panniculitis, and polyarthritis syndrome complicated with terminal pancreatic adenocarcinoma managed with intra‐articular knee aspiration, intra‐articular lidocaine and corticosteroid injection, and decompression of panniculitis: A case report

**DOI:** 10.1002/jgf2.398

**Published:** 2020-11-05

**Authors:** Yuki Takeuchi

**Affiliations:** ^1^ Teine Family Medicine Clinic Sapporo Japan

**Keywords:** family medicine, gastrointestinal medicine, palliative medicine, pancreatitis panniculitis and polyarthritis syndrome, PPP syndrome

## Abstract

Pancreatitis, panniculitis, and polyarthritis (PPP) syndrome is a rare triad of hyperlipasemia, erythematous cutaneous nodules, and oligo‐ or mono‐arthritis and a rare complication of pancreatic diseases. The treatment for PPP syndrome complicated by untreatable pancreatic diseases can be challenging because the causal treatment may not be available. Herein, I report a case of a 72‐year‐old man presenting with PPP syndrome, complicated by untreatable terminal pancreatic adenocarcinoma, who was successfully managed with intra‐articular knee aspiration, intra‐articular injection of lidocaine and corticosteroid, and decompression of panniculitis.

## Graphical abstract

Pancreatitis, panniculitis and polyarthritis (PPP) syndrome complicated with terminal pancreatic cancer.

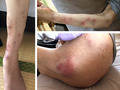

## 1 INTRODUCTION

Pancreatitis, panniculitis, and polyarthritis (PPP) syndrome is a rare triad of hyperlipasemia, erythematous cutaneous nodules, and oligo‐ or mono‐arthritis and a rare complication of pancreatic diseases.^1^, ^2^, ^3^ Common underlying diseases are pancreatitis and pancreatic adenocarcinoma. The causal therapy of PPP syndrome is used for the management of the underlying pancreatic conditions.^3^ Therefore, treatment of PPP syndrome complicated by terminal pancreatic cancer can be challenging, as the causal treatment may not be available. Systemic nonsteroid anti‐inflammatory drugs (NSAIDs) and corticosteroids are often used as symptomatic treatment; however, the efficacy of these drugs is variable.^2^, ^3^ I present a case of PPP syndrome complicated by untreatable terminal pancreatic cancer that did not respond properly to systemic NSAIDs and steroids, but was effectively managed with frequent intra‐articular knee aspiration, intra‐articular injection of lidocaine and corticosteroid, and decompression of panniculitis. The patient in this case provided written, informed consent for publication of the details and the pictures of his condition.

## 2 CASE PRESENTATION

**Figure 1:**
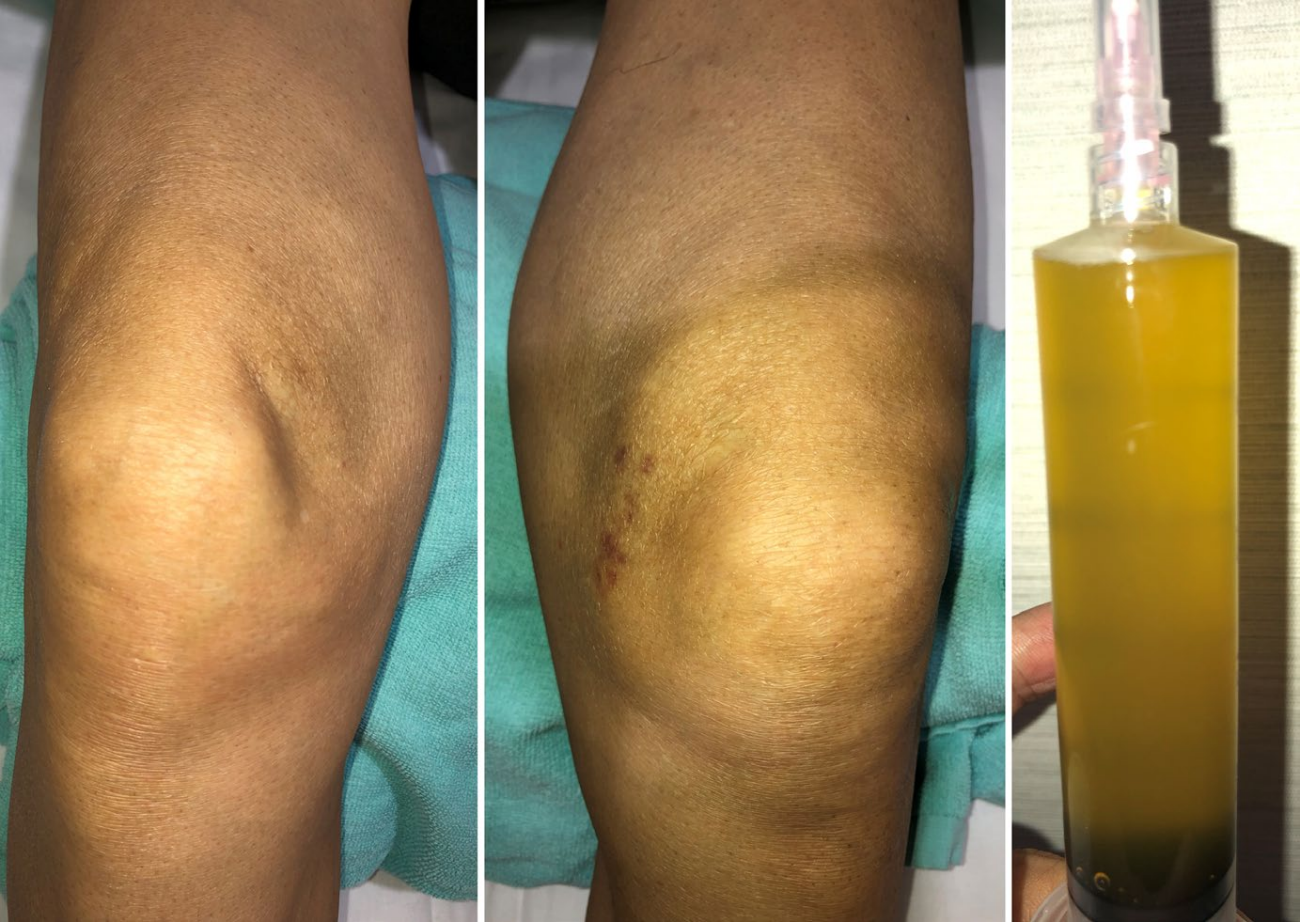
Markedly swollen left knee and cloudy appearance of the synovial fluid [Colour figure can be viewed at wileyonlinelibrary.com]

**Figure 2:**
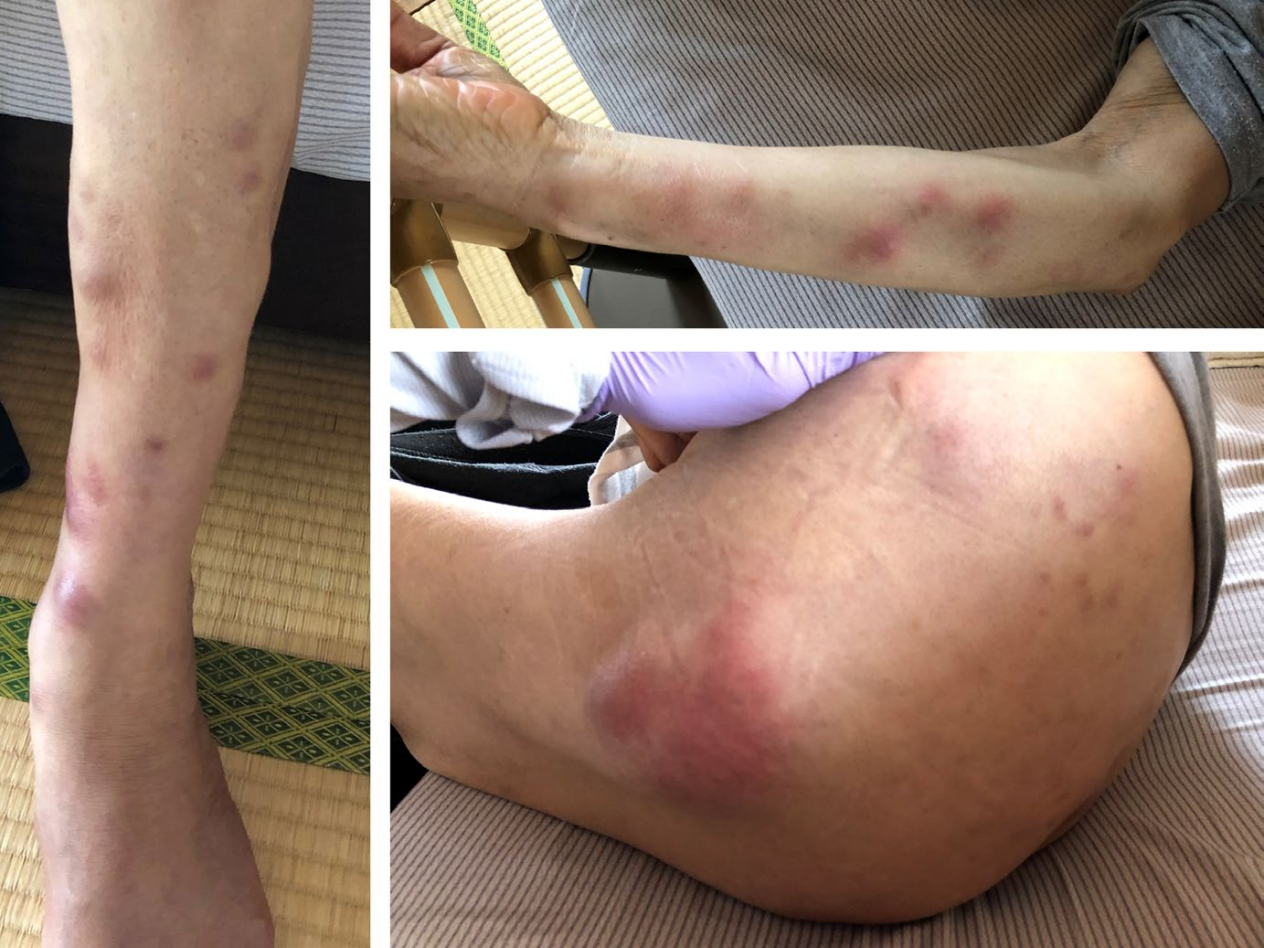
Pancreatic panniculitis on the extremities [Colour figure can be viewed at wileyonlinelibrary.com]

A 72‐year‐old Japanese man with terminal pancreatic adenocarcinoma was referred to my clinic for palliative care after the pancreatic cancer became refractory to chemotherapy. He complained of severe arthralgia in both the knees and multiple painful rashes on the extremities and buttocks. He said, “I have extreme severe burning knee pain. I cannot walk due to the pain despite I have the energy to walk.” The symptoms appeared 3 weeks prior, after the cessation of the chemotherapy, and these symptoms further worsened. His body temperature was 36.2°C, blood pressure was 104/75 mm Hg, pulse rate was 82 beats/min, and oxygen saturation as measured by pulse oximetry was 97% on room air. On physical examination, the left knee was markedly swollen, while the right knee was mildly swollen (Figure 1). He had multiple painful reddish nodules (size 10‐50 mm) on the extremities and buttocks (Figure 2). The blood laboratory test revealed white blood cell count of 9060/µL (normal range, 3300‐8600/µL), C‐reactive protein level of 11.27 mg/dL (normal range, 0.00‐0.14 mg/dL), lipase level of 6847 U/L (normal range, 11‐59 U/L), and thyroid‐stimulating hormone level of 1.04 µIU/mL (normal range, 0.50‐5.00 µIU/mL). The autoimmunity profile (antinuclear antibodies, rheumatoid factor, and anticyclic citrullinated peptide antibody), parvovirus B19 immunoglobulin M antibodies, hepatitis C virus antibodies, and hepatitis B surface antigens were negative. The synovial fluid appeared cloudy (Figure 1). The synovial fluid analysis of the left knee showed cell count of 4150/µL. Bacterial culture of the synovial fluid was negative. Calcium pyrophosphate and uric acid crystals were not detected in the synovial fluid analysis. The radiograph of the knee showed no deformities or joint space narrowing. The skin biopsies of nodules revealed subcutaneous fat necrosis (panniculitis). The diagnosis of PPP syndrome was established based on the presence of subcutaneous fat necrosis and eliminating other causes of arthritis.

At the first visit, the patient was treated with long‐acting oxycodone 20 mg, betamethasone 1 mg, loxoprofen 180 mg, and short‐acting oxycodone 2.5 mg as required. Despite treatment, the patient experienced extremely severe knee pain (numeric rating scale [NRS]: 10/10) and was not able to walk. He was also unable to sleep well because of panniculitis on his buttocks. I gradually increased the dosage of the medications, long‐acting oxycodone 80 mg, betamethasone 4 mg, loxoprofen 180 mg, and short‐acting oxycodone 10 mg as required. However, he still experienced knee pain (NRS: 6‐8/10) and was unable to walk by himself. Although I prescribed him a topical steroid ointment for panniculitis, it was ineffective. I initiated bilateral knee aspiration and intra‐articular knee injection of triamcinolone 40 mg and lidocaine 50 mg. After the procedure, his pain was significantly reduced (NRS: 1‐3/10) and he was able to walk. However, the effect lasted only for 3‐5 days. To alleviate pain, I performed knee aspiration and lidocaine intra‐articular knee injection every week, and triamcinolone intra‐articular injection every month. I introduced an air mattress and automated re‐positioning bed for the decompression of panniculitis. The patient reported that the bed was helpful to reduce pain, and he was able to sleep well. These measures enabled him to walk and sleep well until his overall condition declined because of the progression of pancreatic cancer.

## 3 DISCUSSION

PPP syndrome is a rare complication of pancreatic diseases. Only 1%‐2% of all patients with pancreatic cancer present with pancreatic panniculitis and/or arthritis.^4^ The pathophysiology remains unclear; however, pancreatic enzyme secretion in the bloodstream plays an important role.^5^ Panniculitis is painful erythematous nodule that often emerges on the legs and may spread on the entire body.^3^ Pancreatic polyarthritis is symmetric or asymmetric oligo‐ or mono‐arthritis. Both the large and small joints can be involved. Biopsy of the skin lesion can be an effective diagnostic tool for panniculitis to detect subcutaneous fat necrosis. Although magnetic resonance imaging can be useful to detect intramedullary fat necrosis, no definitive diagnostic tool for pancreatic arthritis is available. Therefore, the other causes of arthritis should be eliminated. The therapy for the underlying pancreatic condition can resolve panniculitis and arthritis.^1^ However, treatment for PPP syndrome with untreatable terminal pancreatic cancer can be difficult. Many authors use systemic NSAIDs and steroid treatment to alleviate symptoms, but the efficacy is often limited.^2^, ^3^ While the patient in this case was not responsive to systemic NSAIDs and corticosteroid, intra‐articular aspiration and injection of corticosteroid and lidocaine for knee pain and decompression for panniculitis reduced the patient's pain significantly and improved the activities of daily living. Although the effect of intra‐articular injection lasted only for 3‐5 days and the patient needed frequent intra‐articular knee aspirations and injections, I believe it was an acceptable measure similar to frequent palliative measure of abdominocentesis.

Pancreatic cancer is the fourth leading cause of cancer death in Japan, and the number of cases is increasing.^6^ Hence, the need for palliative care for pancreatic cancer patients is also increasing. Knowledge regarding PPP syndrome as an important complication of pancreatic cancer is essential to offer optimal treatment. This case shows that intra‐articular aspiration and injection of corticosteroid and lidocaine and decompression of panniculitis can be the palliative treatment options for PPP syndrome.

## CONFLICT OF INTEREST

The authors have stated explicitly that there are no conflicts of interest in connection with this article.

## INFORMED CONSENT

The patient provided informed consent for this case report and the photographic content use. (Figure1.2).
